# Insights into the structural, electronic and magnetic properties of V-doped copper clusters: comparison with pure copper clusters

**DOI:** 10.1038/srep31978

**Published:** 2016-08-18

**Authors:** Dong Die, Ben-Xia Zheng, Lan-Qiong Zhao, Qi-Wen Zhu, Zheng-Quan Zhao

**Affiliations:** 1 School of Science, Xihua University, Chengdu 610039, China; 2 key Laboratory of Advanced Scientific Computation, Xihua University, Chengdu 610039, China

## Abstract

The structural, electronic and magnetic properties of Cu_n+1_ and Cu_n_V (n = 1–12) clusters have been investigated by using density functional theory. The growth behaviors reveal that V atom in low-energy Cu_n_V isomer favors the most highly coordinated position and changes the geometry of the three-dimensional host clusters. The vibrational spectra are predicted and can be used to identify the ground state. The relative stability and chemical activity of the ground states are analyzed through the binding energy per atom, energy second-order difference and energy gap. It is found that that the stability of Cu_n_V (n ≥ 8) is higher than that of Cu_n+1_. The substitution of a V atom for a Cu atom in copper clusters alters the odd-even oscillations of stability and activity of the host clusters. The vertical ionization potential, electron affinity and photoelectron spectrum are calculated and simulated for all of the most stable clusters. Compare with the experimental data, we determine the ground states of pure copper clusters. The magnetism analyses show that the magnetic moments of Cu_n_V clusters are mainly localized on the V atom and decease with the increase of cluster size. The magnetic change is closely related to the charge transfer between V and Cu atoms.

During the last few decades, copper clusters have been demonstrated to have similar catalytic activities with those of gold clusters for the low temperature CO oxidation and partial oxidation of hydrocarbons^1^,^2^,^3^,^4^,^5^,^6^. At the same time, theoretical and experimental work has also shown that the nature of small clusters can be considerably modified by the addition of impurity atom(s)^7^,^8^,^9^,^10^,^11^,^12^,^13^,^14^,^15^,^16^,^17^,^18^,^19^,^20^,^21^,^22^,^23^,^24^,^25^,^26^,^27^,^28^,^29^,^30^,^31^,^32^,^33^,^34^,^35^,^36^,^37^,^38^,^39^. Copper clusters doped with different transition-metal atoms have been expected to tailor the desired catalytic, electronic, magnetic and optical properties for potential applications in solid state chemistry, microelectronics, nanotechnology and materials science^40^,^41^,^42^,^43^,^44^,^45^,^46^,^47^,^48^,^49^,^50^. For instance, Yang *et al.* reported that the adsorption property of copper cluster to CO_2_ can be modified by doping it with Ni atoms and icosahedral Cu_42_Ni_13_ cluster, which is used as catalysts for methanol synthesis via CO_2_ hydrogenation, exhibits the strongest CO_2_ adsorption ability compared to Cu_55_ and Cu_54_Ni clusters^40^. Wang *et al.* found that the melting behavior of Cu-Co bimetallic clusters, which is different from that of pure copper clusters, is closely related to the component materials, stoichiometries and local structure. The Kondo temperature of a Co atom embedded in Cu clusters on Cu(111) exhibits a nonmonotonic variation with the cluster size^41^. Han *et al.* noted that though most of the Cu_n_Ni clusters possess similar geometrics to those of pure copper clusters, Ni-doping introduces a dramatic modulation of the electronic structures, such as the density of states and *d*-band centers^42^. Recently, the Cu-V alloys have investigated due to their unique physical properties. It was shown that the addition of V in Cu alloys can improve mechanical properties and heat resistance of Cu alloys. This effect can be reinforced by increasing the solubility of V in Cu during the synthesis of the alloys^43^. The fcc crystalline structure of Cu-V alloys can be preserved in the solid solution model until the concentration of V reaches a critical value of 23 at.%. When the V concentration in a model is over 23 at.%, a crystal-to-crystal transition will take place^44^. To the best of our knowledge, however, there is a lack of work on small V-doped copper clusters. It has been proved that the gold cluster doped with V can bind a high number of oxygen molecules over pure gold cluster and is an improved novel catalyst for CO oxidation^51^. As it is known, Cu and Au have a similar electronic configurations *nd*^10^(n + 1)*s*^1^. Presumably, the copper clusters doped with V should also be a potential catalyst for the oxidation of CO. On the other hand, some experiments which are used to shed light on the structures of clusters must rely on theoretical calculations of geometries of possible low lying isomers. Therefore, in this paper, the geometric, electronic, and magnetic moments of the small Cu_n+1_ and Cu_n_V (n = 1–12) clusters will be studied systematically on the basis of density functional theory (DFT). It is wished that this work would be helpful to understand the influence of material structure on its properties and could provide practical guidelines for coming experimental research.

## Computational Methods

Geometry optimizations and vibrational frequency analyses of Cu_n+1_ and Cu_n_V clusters have carried out in the framework of a DFT-based method using the GAUSSIAN09 package^52^. The exchange-correlation functional B3LYP and an effective core potential basis set LanL2DZ were used for all of the computations^53^,^54^,^55^,^56^. The convergence thresholds are set to 4.5 × 10^−4^ a.u. for maximum force, 3.0 × 10^−4^ a.u. for root mean square (RMS) force, 1.8 × 10^−3^ a.u. for maximum displacement and 1.2 × 10^−3^ a.u. for RMS displacement. The accuracy of the theoretical level has been checked by calculations on copper dimer and vanadium dimmer. The results have summarized in Table 1. To search the lowest energy structures of Cu_n+1_ and Cu_n_V clusters, lots of initial isomers, which include one-, two- and three-dimensional (3D) configurations, had been taken into account in our geometry optimizations. Owing to the spin polarization, every initial configuration was optimized at possible spin multiplicities. If an imaginary vibraional mode is found, a relaxation of the structure is performed until the true local minimum is actually obtained.

## Results and Discussion

### Geometrical structures and vibrational spectra

**Figure 1:**
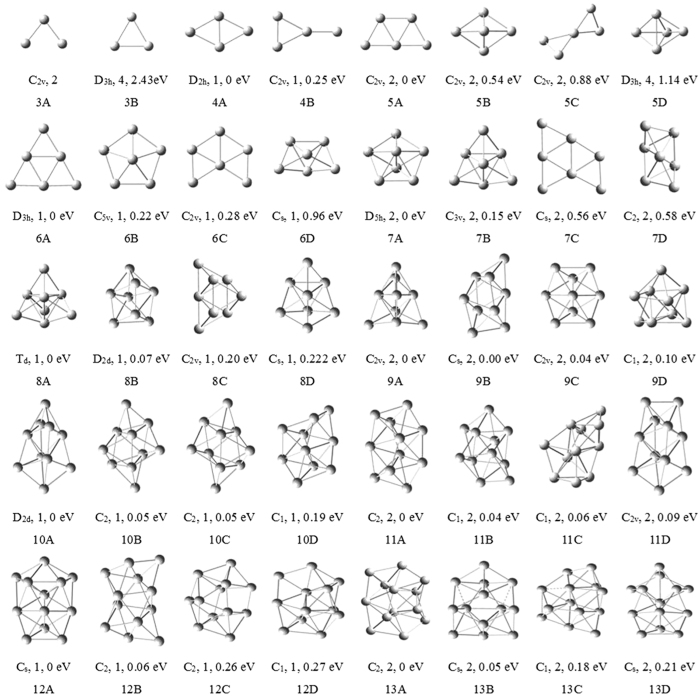
The ground-state structures of Cu_n_ (n = 3–13) clusters, and three low-lying isomers for the Cu_n_ (n = 6–13) clusters. The point group, spin multiplicity, and energy difference compared to each of the ground state structures are given below them.

**Figure 2:**
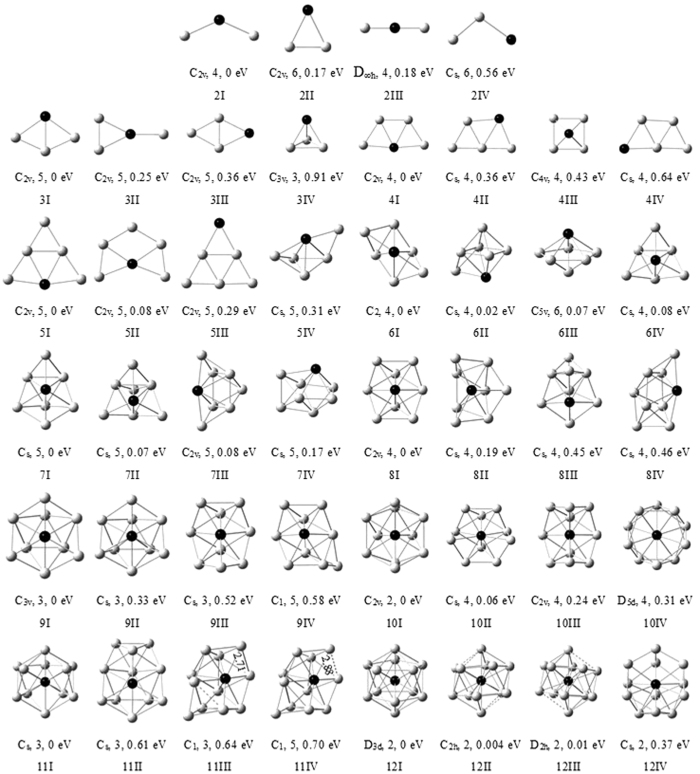
The ground-state structures and three low-lying isomers for CunV (n = 2–12) clusters. The point group, spin multiplicity, and energy difference compared to each of the ground state structures are given below them. The grey and black balls represent Cu and V atoms, respectively.

The optimized results for Cu_2_ and CuV dimmers show the former in singlet spin state is 1.86 eV lower than in triplet spin state and the latter in single, triplet and septet spin states is less stable than in quintet spin state by 2.94, 0.47 and 1.38 eV, respectively. Accordingly, the singlet Cu_2_ and quintet CuV are the ground states. Their bond lengths are 2.26 Å for Cu_2_ and 2.49 Å for CuV. The bond length of the Cu_2_ is shorter than that of the CuV. This may be attributed to the fact that the radius of Cu atom (1.28 Å) is smaller than that of V atom (1.34 Å). For each Cu_n+1_ and Cu_n_V (n = 2–12) clusters, Figs 1 and 2 display the ground state structure and low-lying isomers. According to the energy order from low to high, these isomers are denoted by nA, nB, nC, nD, nI, nII, nIII, and nIV, where n represents the number of Cu atoms in pure copper and Cu_n_V clusters. Meantime, their symmetry, spin multiplicity, and energy difference compared to each of the ground state structures are also indicated in the two figures. The geometric features and mean static polarizabilities (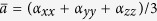) of the ground state Cu_n+1_ and Cu_n_V (n = 1–12) clusters are listed in Table 2.

The most stable structures of Cu_n+1_ and Cu_n_V (n = 2–5) clusters evidently prefer planar configurations. All isomers of copper clusters, which do not include 5C, are found to be in the lowest spin state. The ground state structures of Cu_3_, Cu_4_, Cu_5_ and Cu_6_ clusters are angular, rhombic, trapezoidal and triangular structures, respectively, and no low-lying 3D isomer is obtained for Cu_4_ cluster. When a Cu atom in copper clusters is replaced by one V atom, the number of optimized Cu_n_V structures apparently increases. But only four isomers of each Cu_n_V cluster are depicted in Fig. 2. The lowest energy structures of Cu_2_V, Cu_3_V, Cu_4_V and Cu_5_V clusters is similar to those of Cu_3_, Cu_4_, Cu_5_ and Cu_6_ clusters. The energies of similar structures decrease as the coordination number of V atom increase. The 3IV isomer is the first 3D structure of Cu_n_V clusters. The tetragonal bipyramid and pentagonal pyramid is not unstable for Cu_5_V cluster. Other isomers, which not displayed in Figs 1 and 2, are higher in energy than the nD or nIV isomer.

Starting from n = 6, many stochastic configurations were optimized for Cu_n+1_ and Cu_n_V clusters. The optimized structures show that almost all lower energy isomers possess 3D configurations and V atom in lower energy Cu_n_V cluster tend to occupy the position with the more ligands. As a result, a series of 3D structures for the Cu_n+1_ and Cu_n_V clusters (n = 6–12) were considered and optimized again. Moreover, various 3D Cu_n_V isomers with V atom occupying the most highly coordinated site were optimized further to ensure that the lowest energy structures obtained are the true minimum. To avoid missing the ground state structures, we had also used the strategies of substituting a Cu by one V atom from the pure copper cluster or adding Cu atom(s) to former Cu_n_ or Cu_n_V clusters in geometry optimizations.

The ground state structure of Cu_7_ cluster is a pentagonal bipyramid (7A), lying just below the 7B. The 8A isomer with T_d_ symmetry, which can be treated as a face-capped 7B, is found to be the lowest energy structure of Cu_8_ cluster. The 9A and 9B are nearly degenerate and ref. 57 suggests 9B as the most stable structure. Nevertheless, in view of vertical ionization potential (VIP) which will be discussed later, we deduce that the 9A is the ground state structure of Cu_9_ cluster. Simultaneously, the most stable structures of small Cu_n_ (n = 2–9) clusters had studied by means of optical absorption spectra^58^. Our results are consistent with the previous conclusion. From Cu_10_ to Cu_13_ clusters, the flat cage-like configurations are more stable than other structures, e.g. close-packing and globe-shaped structures. The 10A, 11A, 12A and 13A are the lowest energy structures of Cu_10_, Cu_11_, Cu_12_ and Cu_13_ clusters, respectively. Several isomers reported in ref. 56 have also been optimized at B3LYP/LanL2DZ level and are higher in energy than our lowest energy structures. This is in agreement with Ramirez *et al.’s* studies^57^.

With regard to Cu_n_V (n = 6–12) clusters, the ground state structures (6I, 7I, 8I, 9I, 10I, 11I and 12I) are entirely different from the most stable structure of the corresponding Cu_n+1_ clusters. The 6I, 7I and 8I structures are similar to the low-lying isomers (7D, 8D and 9C) of pure copper clusters. The 9I, 10I, 11I and 12I configurations are unstable or do not exist for Cu clusters in the lowest spin state. The 11I is obtained by distorting the geometry starting from C_5v_ to C_s_ symmetry. The 12I has a small deviation from I_h_ symmetry. The Cu_n_V isomers, which resemble the lowest energy structures and low-lying isomers of Cu_n+1_ clusters, lay above each of the ground state structures (nI). The most stable structures of Cu_n_V (n = 7–12) clusters all contain a pentagonal bipyramid. In addition, due to the Jahn-Teller effect, the 6IV and 7II isomers with C_s_ symmetry have a slight deviation from C_3v_ symmetry. The 10I and 12I strucutres are more stable in doublet spin state than in quartet spin state. The V atom in Cu_n_V clusters tends to occupy the site with the maximum coordination number. This may be ascribed to the principle of maximum overlap in molecular orbital theory. Because the orbital overlap between Cu and V atoms increases, the energy of Cu_n_V cluster will decrease.

**Figure 3:**
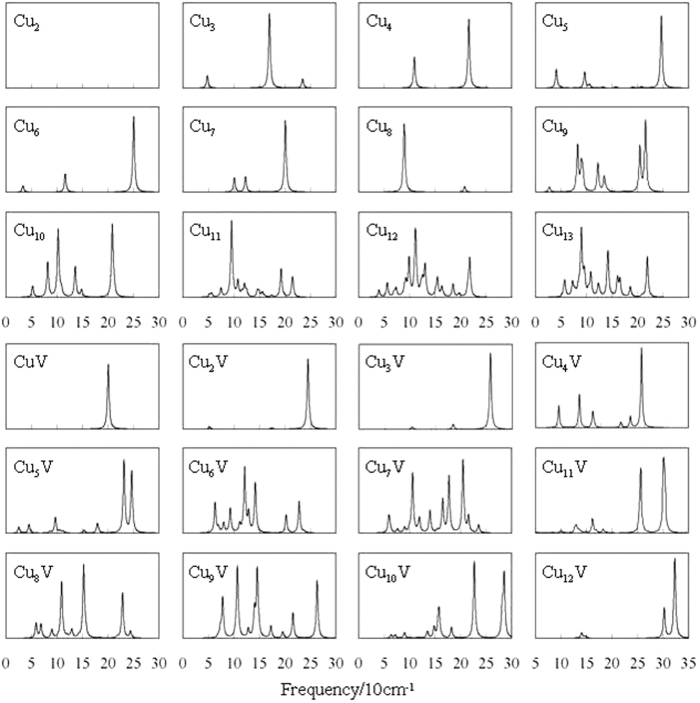
Vibrational spectra of the ground state Cu_n+1_ of Cu_n_V (n = 1–12) clusters.

The combination of theoretical and experimental vibrational spectra is a good method for the structural determination of small isolated clusters and the method has been successfully applied in practice^59^. Consequently, the vibrational spectra of the lowest energy Cu_n+1_ and Cu_n_V (n = 1–12) clusters are shown in Fig. 3. The Cu_2_ dimer merely has a stretching vibration without change of dipole moment, so there is no absorption peak. The absorbed peaks of planar or highly symmetrical clusters are less than those of other configuration clusters. The intense peaks of 3D Cu_n_V (n = 6–12) clusters are more than those of corresponding pure Cu_n+1_ clusters. The vibrational fundamentals of all Cu_n+1_ and Cu_n_V clusters are found to be in the range of 5 to 330 *cm*^−1^. The most intense peak of vibrational spectum of each Cu_n_V clusters is related to the V-Cu stretching vibrations. The characteristic frequencies of the ground state structures and several low-lying isomers are given as Supplementary Material.

### Relative stabilities and electronic properties

In this part, the relative stabilities and electronic properties of the ground state Cu_n+1_ and Cu_n_V (n = 1–12) clusters are discussed by means of the atomic averaged binding energies, second-order energy differences, energy gaps between the highest occupied molecular orbital (HOMO) and lowest unoccupied molecular orbital (LUMO), the VIP, the vertical electron affinity (VEA) and photoelectron spectroscopy (PES).

**Figure 4:**
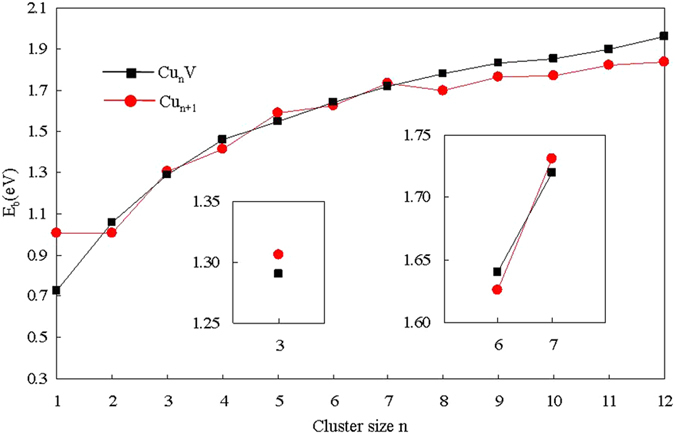
Size dependence of the averaged binding energies for the ground state Cu_n+1_ and Cu_n_V (n = 1–12) clusters.

The atomic averaged binding energies (*E*_*B*_) of the Cu_n+1_ and Cu_n_V clusters can be calculated as follows


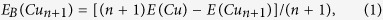



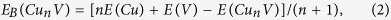


where *E*(*Cu*_n+1_), *E*(*Cu*), *E*(*Cu*_*n*_*V*) and *E*(*V*) are the energy of Cu_n+1_ cluster, Cu atom, Cu_n_V cluster and V atom, respectively. The calculated binding energies per atom for the lowest energy Cu_n+1_ and Cu_n_V clusters are shown in Fig. 4. As seen from this figure, the size dependence of *E*_*B*_ for Cu_n+1_ clusters have an apparent peak at n = 7. That is to say, the Cu_8_ cluster possesses relatively higher thermic stability. The *E*_*B*_ of Cu_n_V clusters, which is larger than that of Cu_n+1_ clusters for n≥8, is a monotonically increasing function of the number of atoms in clusters. This implies that the doped clusters can continue to gain energy during growth process. The substitution of a V atom for a Cu atom in Cu_n+1_ (n≥8) clusters can evidently enhance the stability of the host clusters. The phenomenon may be caused by structural changes. The configuration of Cu_n_V (n≥8) clusters is entirely different from that of Cu_n+1_ clusters.

**Figure 5:**
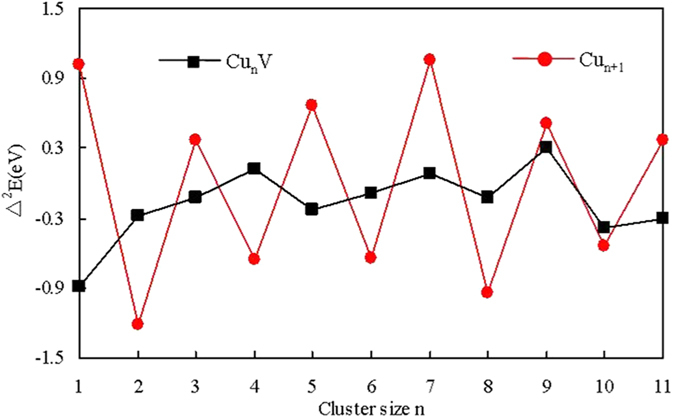
Size dependence of the second-order energy differences for the lowest energy Cu_n+1_ and Cu_n_V (n = 1–12) clusters.

In cluster physics, the second-order energy differences (*Δ*^2^*E*), which can be compared with the relative abundances determined in mass spectroscopy experiment, is a particularly sensitive quantity that reflects the relative stability of clusters. For the ground state Cu_n+1_ and Cu_n_V clusters, it can be calculated as


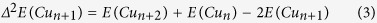



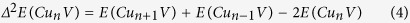


where *E* is the energy of the ground-state clusters. The calculated second-order energy differences as a function of the cluster size are illustrated in Fig. 5. It is obvious from Fig. 5 that the even-numbered copper clusters are more stable than the odd-numbered ones. However, the introduction of a V atom in copper cluster alters the stable pattern of the host clusters significantly. For the Cu_n_V clusters, three maxima are observed at n  = 4, 7 and 9. Accordingly, it can be inferred that the Cu_4_V, Cu_7_V and Cu_9_V clusters are magic clusters and have an enhanced abundance in mass spectra.

**Figure 6:**
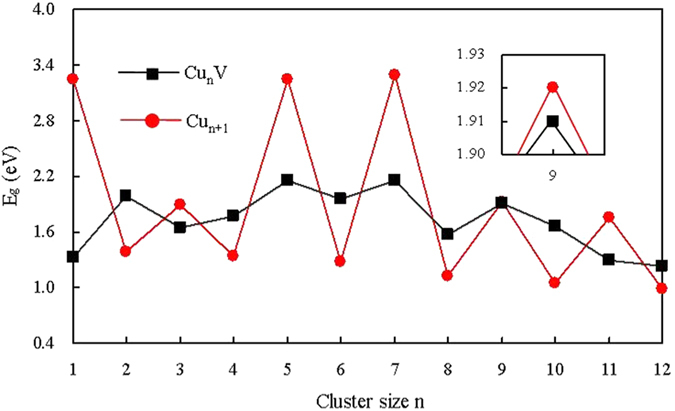
Size dependence of the HOMO-LUMO energy gaps of the most stable Cu_n+1_ and Cu_n_V (n = 1–12) clusters.

The HOMO-LUMO energy gap (*E*_g_), which relies on the eigenvalues of the HOMO and LUMO energy levels, is viewed as an important parameter that characterizes chemical stability of small clusters. A big energy gap usually relates to a high chemical inertness. For the ground state Cu_n+1_ and Cu_n_V clusters, the energy gaps are plotted in Fig. 6. The pure copper clusters show an odd-even alternation in their energy gaps. This phenomenon can be interpreted by the electron pairing effect that the electron in a doubly occupied HOMO has stronger effective core potentials because the electron screening is weaker for electrons in the same orbital than for inner shell electrons. When a Cu atom ([Ar]3*d*^10^4*s*^1^) in Cu_n+1_ cluster is replaced by a V ([Ar]3*d*^3^4*s*^2^) atom, the closed electronic shell will become an opened electronic shell. So, the *E*_g_ of Cu_n_V cluster for n = odd is smaller than that of Cu_n+1_ cluster. For n = 2, 4, 6 and 8, the unpaired electrons of Cu_n_V cluster is more than those of the corresponding Cu_n+1_ cluster. The energy of the LUMO of Cu_n_V cluster will rise because of the electrostatic interaction of unpaired electrons. The *E*_g_ of Cu_n_V are larger than that of the Cu_n+1_ cluster. For n = 10 and 12, the Cu_n_V cluster is equal to Cu_n+1_ cluster in unpaired electrons. However, the formers have a highly symmetrical geometry. Hereby, the *E*_g_ of Cu_10_V and Cu_12_V clusters is also larger than that of the Cu_11_ and Cu_13_ clusters, respectively.

**Figure 7:**
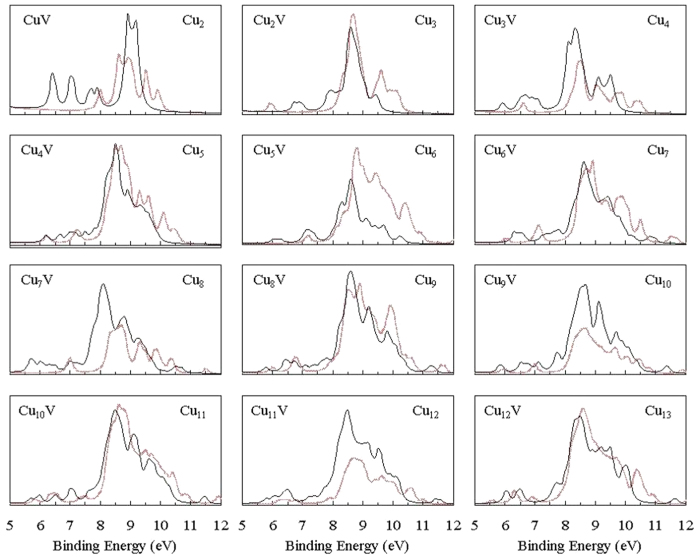
Simulated photoelectron spectra of the ground state Cu_n+1_ (in red) and Cu_n_V (in black) (n = 1–12) clusters.

The VIP and VEA are two basic quantities to get an insight into the electronic property and can be estimated as follows


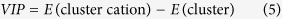



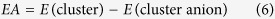


where *E*(cluster cation) and *E*(cluster anion) are the single-point energies of the cationic and anionic clusters in the neutral geometry. For the lowest energy Cu_n+1_ and Cu_n_V clusters, Table 3 give the calculated VIP and VEA along with the available experimental data. The calculated VIPs of pure copper clusters are in good agreement with previous measurements obtained at discrete 2.5 nm intervals. The agreement confirmed reliability of the present theoretical method again. Meanwhile, we can distinguish the ground state structure of Cu_9_ cluster by the aid of VIPs. The present and preceding calculations have shown that the 9A and 9B isomers are the candidate for the lowest energy structure of Cu_9_ cluster. Our calculated VIPs are 5.27 eV for 9A and 5.99 eV for 9B. The measured value is 5.36 ± 0.05 eV^60^. Thus, we deduced that the 9A structure is the most stable structure of Cu_9_ cluster. To offer reference material for PES experiment in future, the theoretical PES spectra of the global minimum structures of Cu_n+1_ and Cu_n_V (n = 1–12) clusters were simulated by adding the occupied orbital energy relative to the HOMO to the VIP and fitting them with a broadening factor of 0.1 eV, as plotted in Fig. 7. The distribution of energy level for all clusters is in the range of 6 to 11 eV. The doped V atom made a change for the PES spectra of copper cluster. This change is relatively pronounced for Cu_n+1_ and Cu_n_V (n = 1–5) clusters. The pronounced change might be related with the planar configuration.

### Magnetic properties

**Figure 8:**
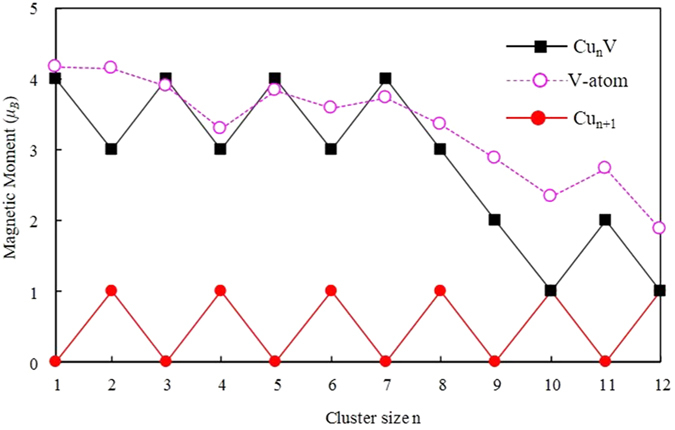
Total magnetic moment of the ground state Cu_n+1_ and Cu_n_V (n = 1–12) clusters and local magnetic moment on the dopant atom V.

**Figure 9:**
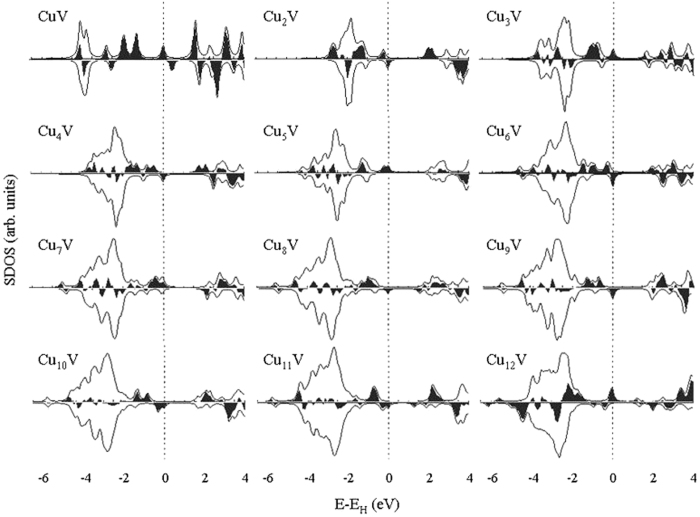
SDOS of the lowest energy Cu_n_V clusters. A broadening factor δ = 0.1 eV is used. Spin up (positive) and spin down (negative) densities are given in each case. The black part is the density difference (spin up minus spin down). The dashed line indicates the location of the HOMO level.

The magnetic properties of the clusters are not only widely used in the preparation of nano electronic devices and high density magnetic storage materials, but also have a very important theoretical significance in the basic research of physics. The total magnetic moments of cluster mainly include the orbital and spin magnetic moments of electrons. The orbital magnetic moment of an electron is far less than the spin magnetic moment and, consequently, the magnetic moment of cluster is dominated by the spin magnetic moment. For the ground-state Cu_n+1_ and Cu_n_V (n = 1–12) clusters, the total magnetic moments are calculated and displayed in Fig. 8. The lowest energy copper clusters show an odd–even alternations with the increase of Cu atom in the total magnetic moment. The magnetic moment of Cu_n+1_ clusters with odd n is completely quenched. For the doped clusters, the magnetic moment of Cu_n_V (n = 1–8) cluster is far larger than that of Cu_n+1_ clusters. The substitution of a V atom for a Cu atom can enhance the magnetism of the small host cluster. The Cu_2_V, Cu_4_V, Cu_6_V and Cu_8_V clusters have a magnetic moment of 3 *μ*_B_, which is also the magnetic moment of a V atom. The magnetic moment (4 *μ*_B_) of each Cu_n_V (n = 1, 3, 5 and 7) clusters is just equal to the sum of the magnetic moments of the Cu_n_ cluster (1 *μ*_B_) and an isolated V atom (3 *μ*_B_). These imply that the interaction of Cu and V atoms may have similarities among Cu_n_V (n = 1–8) clusters. In case of big Cu_n_V (n = 9–12) cluster, the Cu_10_V and Cu_12_V clusters have the same magnetic moments as Cu_11_ and Cu_13_ clusters. The magnetic moment (2 *μ*_B_) of Cu_9_V and Cu_11_V clusters be greater than that (1 *μ*_B_) of Cu_9_ and Cu_11_ clusters and less than that (3 *μ*_B_) of V atom. The foregoing relation indicates that the big Cu_n_V (n = 9–12) clusters have a different interaction between Cu and V atoms relative to Cu_n_V (n = 1–8) clusters. As an effort to explain the magnetism, Fig. 9 gives the spin density of states (SDOS) for the global minimum structures of Cu_n+1_ and Cu_n_V clusters. All the ground states have an intense band between −5 and −2 eV, which consists principally of the valence *s* and *d* orbitals of the constituent atoms. It is clear from the density difference that the magnetic moment of Cu_n_V clusters mostly comes from the electrons near the HOMO (*E*−*E*_*H*_ = −2~0 *eV*). The Cu_n_V clusters have some very small magnetic domains, which vary with the size of cluster.

**Figure 10:**
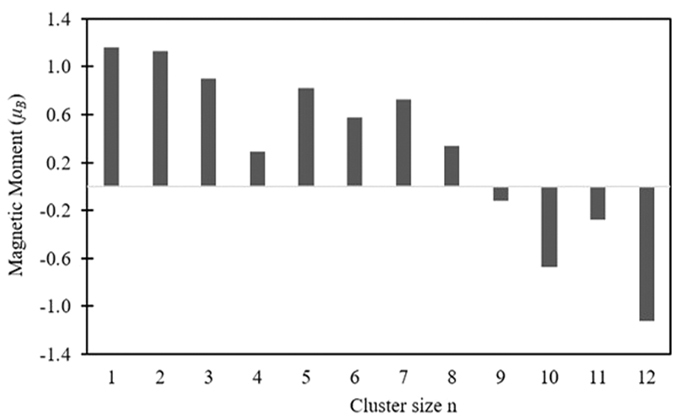
Free V atom as the reference point, the change of magnetic moments of V atom in Cu_n_V clusters.

**Figure 11:**
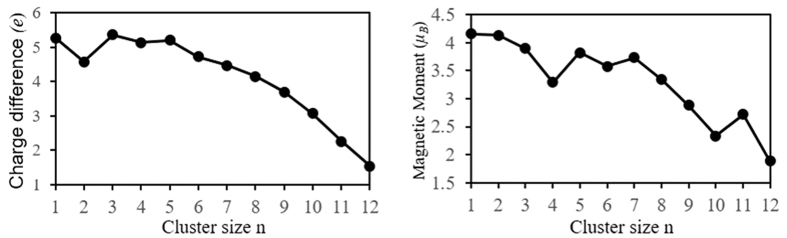
The relation between the charge and magnetic moment of V atom in Cu_n_V clusters.

To gain insight into the magnetic properties further, we have performed the natural bond orbital analysis^61^ for the lowest energy Cu_n_V clusters. The local magnetic moments on V atom are 4.16 *μ*_B_ for CuV, 4.13 *μ*_B_ for Cu_2_V, 3.90 *μ*_B_ for Cu_3_V, 3.29 *μ*_B_ for Cu_4_V, 3.82 *μ*_B_ for Cu_5_V, 3.58 *μ*_B_ for Cu_6_V, 3.73 *μ*_B_ for Cu_7_V, 3.34 *μ*_B_ for Cu_8_V, 2.88 *μ*_B_ for Cu_9_V, 2.33 *μ*_B_ for Cu_10_V, 2.72 *μ*_B_ for Cu_11_V and 1.88 *μ*_B_ for Cu1_2_V, as shown in Fig. 8. Overall, with the increase of cluster size, the magnetic moments of V atoms gradually decrease. The magnetic moments of V atom in Cu_n_V clusters is larger for n = 1–8 and smaller for n = 9–12 than that of free V atom. Compared to the free V atom, the change of magnetic moments of V atoms in Cu_n_V clusters (see Fig. 10) should reflect the strength of the interaction between V and Cu atoms. The magnetic moment provided by Cu atoms is very small. Furthermore, Cu atoms in Cu_n_V (n = 1, 9, 11 and even) clusters exhibit an antiferromagetic alignment with respect to the V atom’s magnetic moment. That is to say, the magnetic moments of these Cu_n_V clusters primarily are from a paramagnetic V atom. The charge and magnetic moment on 4*s*, 3*d*, 4*p* and 5*p* orbitals of V atom in Cu_n_V clusters are listed in Table 4. It can be seen from the table that the partially filled 3*d* orbital play a substantial role in determining the magnetism of V atom. The magnetic moment of 3*d* orbital is 1.81~3.98 *μ*_B_. The 4*s* and 4*p* orbitals, which are non-magnetic for a free V atom, contribute a few of magnetic moment, apart from 4*p* orbital of V in CuV dimer. This may be ascribed to the internal charge transfer from 4s to 3*d*, 4*p* and 5*p* orbitals. Simultaneously, there are an interatomic charge transfers in Cu_n_V clusters. Namely, 0.13–0.36 electrons transfer from V atom to Cu atoms for n = 1, 3–5 and 0.28–3.46 electrons from Cu atoms to V atom for n = 2, 6–12. As we know, the *d* orbital can contain up to 10 electrons. If *N* represents the sum of valence electron on V atom in Cu_n_V clusters, we found that 10-*N* and the magnetic moment of V atom have the same change trend, as shown in Fig. 11. The charge transfer hints that the V atom in Cu_n_V clusters has a hybridization among *s*, *p* and *d* orbitals. The energy of *d* orbital of V atom is gradually decreased with the increase of clusters size and more and more electrons are transferred to the *d* orbital. Hence, the larger the cluster, the smaller the magnetic moment of V atom. The orbital hybridization and charge transfer should be responsible for the magnetic moment alteration of the dopant atom.

## Conclusions

Density functional calculations have been performed for the structural, electronic, and magnetic properties of Cu_n+1_ and Cu_n_V (n = 1–12) clusters. The results show that V atom in low energy Cu_n_V clusters tend to occupy the position with the maximum coordination number and changes the geometry of the 3D host clusters. The vibrational and photoelectron spectroscopy spectra are given to identify the most stable structures in times to come. The substitution of a Cu atom in copper clusters by a V atom enhances the binding energy of big clusters and alters the odd-even oscillations of relative stability and chemical activity of the host clusters. The ground states of copper clusters are confirmed by comparing the theoretical vertical ionization potential with experimental findings. At the same time, we predict the vertical ionization potential and electron affinity of Cu_n_V clusters and electron affinity of Cu_n+1_ cluster. The magnetism calculation indicates that V atom in Cu_n_V clusters carries most of the total magnetic moment. The local magnetic moment of the doped atom decreases with the increase of cluster size because of the orbital hybridization and charge transfer.

## Additional Information

**How to cite this article**: Die, D. *et al.* Insights into the structural, electronic and magnetic properties of V-doped copper clusters: comparison with pure copper clusters. *Sci. Rep.* **6**, 31978; doi: 10.1038/srep31978 (2016).

## Supplementary Material

Supplementary Information

## Figures and Tables

**Table 1:** The geometries and electronic properties of Cu_2_ and V_2_ dimers.

Dimer	Functional/Basis set	*r*(Å)		D_e_(eV)		*f*(cm^−1^)		VIP(eV)		EA(eV)	
		Calc.	Expt.	Calc.	Expt.	Calc.	Expt.	Calc.	Expt.	Calc.	Expt.
Cu_2_	B3LYP/LanL2DZ	2.26	2.22^a^	2.02	2.01^a^	256	264^a^	7.99	7.90^a^	0.63	0.83^a^
	BLYP/LanL2DZ	2.25		2.28		264		8.06		0.62	
	PW91/LanL2DZ	2.23	j	2.35		273		8.09		0.72	
	B3LYP/6-311 + G(d)	2.28		1.80		239		8.07		0.81	
	Blyp/6-311 + G(d)	2.27		2.01		244		8.20		0.83	
	PW91/6-311 + G(d)	2.24		2.11		260		8.22		0.93	
V_2_	B3LYP/LanL2DZ	1.75	1.77^a^	1.94	2.47 ± 0.22^a^			6.39	6.35^a^		

^a^Refs 62, 63, 64, 65.

**Table 2:** The maximum and minimum bond lengths (R_max_, R_min_) and chemical bond per atom (C) for the most stable Cu_n+1_ and Cu_n_V clusters.

Clusters	R_max_ (Å)	R_min_ (Å)	C	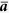 (a.u.)	Clusters	R_max_ (Å)	R_min_ (Å)	C	N	R_v_ (Å)	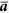 (a.u.)
Cu_2_	2.26	2.26	0.5	0	CuV	2.49	2.49	0.5	1	2.49	0.04
Cu_3_	2.33	2.33	0.7	8.60	Cu_2_V	2.47	2.47	0.7	2	2.47	2.78
Cu_4_	2.45	2.31	1.3	4.28	Cu_3_V	2.67	2.40	1.3	3	2.63	12.25
Cu_5_	2.47	2.41.	1.4	2.68	Cu_4_V	2.59	2.40	1.4	4	2.58	3.88
Cu_6_	2.5	2.41	1.5	7.73	Cu_5_V	2.68	2.42	1.5	4	2.64	7.56
Cu_7_	2.67	2.50	2.3	1.31	Cu_6_V	2.65	2.40	2.1	6	2.61	2.08
Cu_8_	2.56	2.49	2.3	1.92	Cu_7_V	2.65	2.46	2.3	6	2.64	2.50
Cu_9_	2.64	2.46	2.3	1.43	Cu_8_V	2.74	2.54	2.6	8	2.62	2.50
Cu_10_	2.63	2.48	2.5	3.98	Cu_9_V	2.64	2.47	2.7	9	2.59	2.34
Cu_11_	2.64	2.48	2.6	3.44	Cu_10_V	2.66	2.47	2.8	10	2.57	1.81
Cu_12_	2.65	2.47	2.6	4.12	Cu_11_V	2.71	2.50	2.6	11	2.54	2.42
Cu_13_	2.68	2.47	2.8	2.72	Cu_12_V	2.67	2.51	2.8	12	2.51	3.17

The averaged bond length between V and Cu atoms (R_v_) and coordination number (N) of V atom in the ground state Cu_n_V clusters. The mean static polarizabilities (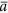).

**Table 3:** VIP and EA of the most stable Cu_n+1_ and Cu_n_V clusters.

Clusters	VIP(eV) Calc.	VIP(eV) Expt.	EA(eV) Calc.	Clusters	VIP(eV) Calc.	EA(eV) Calc.
Cu_2_	7.99	7.90^a^	0.63	CuV	6.43	0.77
Cu_3_	5.94	5.80 ± 0.05^a^	1.05	Cu_2_V	6.73	1.18
Cu_4_	6.60	7.15 ± 0.75^a^	1.27	Cu_3_V	5.93	1.21
Cu_5_	6.24	6.30 ± 0.05^a^	1.70	Cu_4_V	6.20	1.41
Cu_6_	7.17	7.15 ± 0.75^a^	0.96	Cu_5_V	6.04	1.05
Cu_7_	6.04	6.07 ± 0.05^a^	1.68	Cu_6_V	6.32	1.55
Cu_8_	6.98	7.15 ± 0.75^a^	0.92	Cu_7_V	5.66	1.15
Cu_9_	5.27	5.36 ± 0.05^a^	1.43	Cu_8_V	5.78	1.40
Cu_10_	5.95	6.07 ± 0.05^a^	1.29	Cu_9_V	5.85	1.56
Cu_11_	5.84	5.91 ± 0.05^a^	2.11	Cu_10_V	5.69	1.85
Cu_12_	6.26	6.30 ± 0.05^a^	1.76	Cu_11_V	5.79	1.86
Cu_13_	5.57	5.66 ± 0.05^a^	2.07	Cu_12_V	6.02	2.10

^a^Refs 60 and 66.

**Table 4:** The charge (Q) and local magnetic moment (M) of 3d, 4s, 4p, and 5p states for the V atom in the ground state Cu_n_V clusters.

Clusters	V-4s		V-3d		V-4p		V-5p	
	Q (e)	M (μ_B_)	Q (e)	M (μ_B_)	Q (e)	M (μ_B_)	Q (e)	M (μ_B_)
CuV	0.66	0.18	4.06	3.98	0.02	0	0	0
Cu_2_V	1.38	0.58	3.71	3.45	0.34	0.10	0	0
Cu_3_V	0.49	0.17	3.85	3.65	0.3	0.08	0	0
Cu_4_V	0.59	0.01	3.73	3.27	0.55	0.01	0	0
Cu_5_V	0.56	0.1	3.91	3.67	0.33	0.05	0	0
Cu_6_V	0.47	0.01	3.91	3.19	0.73	0.21	0.17	0.17
Cu_7_V	0.48	0.08	4.12	3.54	0.93	0.11	0	0
Cu_8_V	0.47	0.05	4.23	3.19	1.16	0.10	0	0
Cu_9_V	0.45	0.03	4.43	2.77	0.35	0.01	1.07	0.07
Cu_10_V	0.46	0.02	4.68	2.26	1.16	0.02	0.63	0.03
Cu_11_V	0.47	0.03	5.00	2.58	2.27	0.11	0	0
Cu_12_V	0.46	0.02	5.41	1.81	2.59	0.05	0	0
