# Supplementary material for: Insights into the structural, electronic and magnetic properties of V-doped copper clusters: comparison with pure copper clusters
Source: Sci Rep. 2016 Aug 18;6:31978. doi: 10.1038/srep31978 (PMC4989221; doi:10.1038/srep31978)
Supplement: Supplementary Information [file srep31978-s1.docx]

**Insights into the structural, electronic and magnetic properties of V-doped copper clusters: comparison with pure copper clusters**

Dong Die^1,2^, Ben-Xia Zheng^1,^^[[1]](#footnote-1)^#, Lan-Qiong Zhao^1^, Qi-Wen Zhu^1^ & Zheng-Quan Zhao^1^

| Clusters | *f*_n-A_ | *f*_n-B_ | *f*_n-C_ | *f*_n-D_ | Clusters | *f*_n-I_ | *f*_n-II_ | *f*_n-III_ | *f*_n-IV_ |
| --- | --- | --- | --- | --- | --- | --- | --- | --- | --- |
| Cu_2_ | 256 |  |  |  | CuV | 201 |  |  |  |
| Cu_3_ | 170 | 84 |  |  | Cu_2_V | 245 | 59 | 262 | 219 |
| Cu_4_ | 216 | 231 |  |  | Cu_3_V | 257 | 255 | 202 | 232 |
| Cu_5_ | 247 | 105 | 143 | 199 | Cu_4_V | 243 | 250 | 244 | 241 |
| Cu_6_ | 250 | 93 | 171 | 112 | Cu_5_V | 231 | 185 | 230 | 241 |
| Cu_7_ | 201 | 122 | 236 | 182 | Cu_6_V | 121 | 208 | 135 | 138 |
| Cu_8_ | 89 | 215 | 106 | 200 | Cu_7_V | 205 | 131 | 87 | 161 |
| Cu_9_ | 216 | 220 | 87 | 202 | Cu_8_V | 152 | 210 | 213 | 212 |
| Cu_10_ | 208 | 198 | 200 | 212 | Cu_9_V | 145 | 108 | 230 | 199 |
| Cu_11_ | 96 | 205 | 218 | 124 | Cu_10_V | 227 | 226 | 234 | 215 |
| Cu_12_ | 111 | 122 | 121 | 122 | Cu_11_V | 300 | 101 | 281 | 282 |
| Cu_13_ | 90 | 219 | 215 | 100 | Cu_12_V | 322 | 324 | 321 | 235 |

S1. The characteristic frequency (cm^-1^) of Cu_n+1_ and Cu_n_V clusters.

1. ^1^School of Science, Xihua University, Chengdu 610039, China. ^2^key Laboratory of Advanced Scientific Computation, Xihua University, Chengdu 610039, China. Correspondence and requests for materials should be addressed to D.D (email: science_dd@163.com). #This author contributed equally to this work and should be considered co-first author [↑](#footnote-ref-1)
